# Image Correction Methods for Regions of Interest in Liver Cirrhosis Classification on CNNs

**DOI:** 10.3390/s22093378

**Published:** 2022-04-28

**Authors:** Yoshihiro Mitani, Robert B. Fisher, Yusuke Fujita, Yoshihiko Hamamoto, Isao Sakaida

**Affiliations:** 1 National Institute of Technology, Ube College, Ube 755-8555, Japan; 2 School of Informatics, The University of Edinburgh, Edinburgh EH8 9BT, UK; r.b.fisher@ed.ac.uk; 3 Faculty of Engineering, Yamaguchi University, Ube 755-8611, Japan; y-fujita@yamaguchi-u.ac.jp (Y.F.); hamamoto@yamaguchi-u.ac.jp (Y.H.); 4 School of Medicine and Health Sciences, Yamaguchi University, Ube 755-8505, Japan; sakaida@yamaguchi-u.ac.jp

**Keywords:** B-mode ultrasound images, liver cirrhosis classification, convolution neural networks, image correction, image quality improvement, inverse of tone curves, tone curves

## Abstract

The average error rate in liver cirrhosis classification on B-mode ultrasound images using the traditional pattern recognition approach is still too high. In order to improve the liver cirrhosis classification performance, image correction methods and a convolution neural network (CNN) approach are focused on. The impact of image correction methods on region of interest (ROI) images that are input into the CNN for the purpose of classifying liver cirrhosis based on data from B-mode ultrasound images is investigated. In this paper, image correction methods based on tone curves are developed. The experimental results show positive benefits from the image correction methods by improving the image quality of ROI images. By enhancing the image contrast of ROI images, the image quality improves and thus the generalization ability of the CNN also improves.

## 1. Introduction

In the medical imaging field, a computer-aided diagnosis (CAD) (e.g., [1,2,3]) system which gives a second opinion is strongly needed. Ultrasound imaging is non-invasive and widely used for the diagnosis of liver cirrhosis [4]. Cirrhosis of the liver is expected to progress to liver cancer in the worst case. Therefore, we are investigating a CAD system to diagnose liver cirrhosis sooner [5,6]. Many believe that using machine learning and artificial intelligence is effective for designing CAD systems. In general, there are two types of ultrasound images: B-mode and M-mode. The B-mode shows the whole of the breast imaging, including the liver. On the other hand, the M-mode provides motion images of the liver aortic vessels [7].

**Figure 1:**
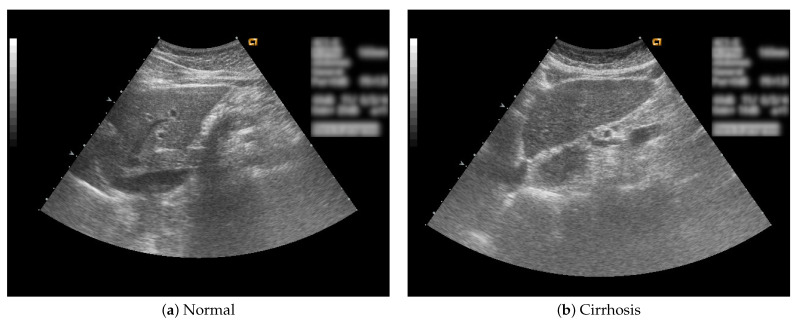
Liver ultrasound images.

**Figure 2:**
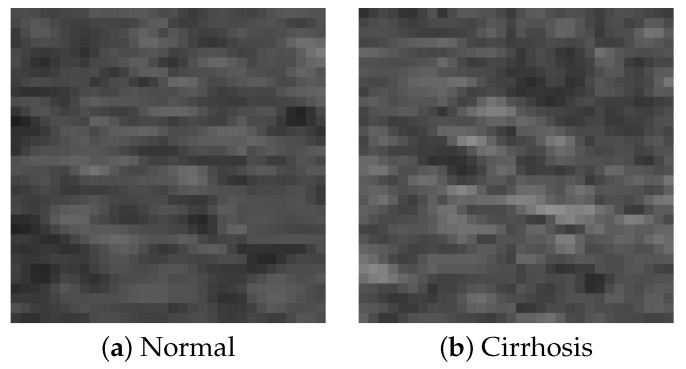
ROI images.

In this study, B-mode liver ultrasound imaging is focused on. B-mode ultrasound image data was provided by the collaborator. These data include 12 cirrhosis patients and 8 normal subjects. There were five ultrasound images per person. Figure 1 shows B-mode liver ultrasound images. Figure 1a,b show a normal and a cirrhosis liver, respectively. In this study, we focus on classifying regions of interest (ROIs) from the B-mode ultrasound images. Figure 2 shows examples of ROI images. The ROI images were manually cut out from the liver areas by a physician. A total of 200 normal and 300 cirrhosis ROI images were collected. Figure 2a,b show normal and cirrhosis livers. The size of each ROI image was 32 by 32 pixels. The grey level was 8 bits. Thus, the values ranged from 0 to 255. This is a typical two-class problem, normal or cirrhosis. From this figure, it seems difficult to visually classify a liver as normal or cirrhosis if one is not a physician because of noisy ultrasound images.

In a previous study [5], we explored liver cirrhosis classification using an approach based on higher-order local auto-correlation (HLAC) features, which are known as hand-crafted features in the pattern recognition field. The HLAC feature approach produced the best performance among our experimental results. However, this experimental result showed that the average error rate was over 40%. Even the best performance from the conventional approach was not very good.

Convolution neural networks (CNNs) are widely used in the medical imaging field (e.g., [8]). In recent studies, as shown in (e.g., [9,10,11]), issues of liver diseases as captured in ultrasound images have been addressed. Deep learning approaches were adopted in [9,11], and a combination of hand-crafted features and the classical classifier was used in [10]. Each of these approaches was reported to result in better accuracy. Note, however, that the datasets themselves were all different. Each of the datasets used in [9,11] was for liver fibrosis staging classification. The dataset in [11] was for normal and fatty liver, not cirrhosis. On the other hand, the dataset we used was for normal and cirrhosis. Cirrhosis is the most dangerous liver disease, as mentioned above. Therefore, it is difficult to collect a sufficient amount of data on it. The limitation on the available cirrhosis data increases the difficultly of the pattern recognition problem. The average error rate on the limited dataset may thus be high compared to another type of dataset.

We expect that the deep neural nets will be able to yield a good performance in this liver cirrhosis classification problem. One defect of CNNs is the need for many training samples. This leads to an over-training problem. In a previous study [6], we showed the effectiveness of the ROI image augmentation method by a perspective transformation.

Apart from the ROI image data augmentation, we would also like to improve the average error rate of liver cirrhosis classification using CNNs. By contrast with the conventional pattern recognition system, the CNN could work well by learning a combination of input images and output labels or class names. Thus, we may overlook the importance of obtaining better image quality. In the pattern recognition field in general, the richer the features that are obtained, the better the classification performance of the pattern recognition system. Most classifiers work well only if the features are good. Therefore, there has always been much effort devoted to obtaining better features, with both the classical and the deep learning approaches. However, deep learning can reduce the efforts because the CNN learns automatically by feeding up a combination of input images and output labels. Therefore, our focus is on improving the image quality of ROI images with the goal of improving the classification of liver cirrhosis. The method called CEUS (contrast-enhanced ultrasound) (e.g., [12]) has been expected to obtain more contrast-enhanced images than conventional ultrasound imaging. In this study, on the other hand, the aim is to apply image processing to the already available images to obtain higher-quality images.

Livers with cirrhosis are known to be physically harder. This would make the image regions slightly lighter. On the other hand, a normal liver is not so hard. In this case, the image regions are slightly darker. Therefore, we expect that the image contrast of ROI images is one of the important factors in classifying liver cirrhosis. In order to highlight the image contrast of ROI images, we adopted the approach of enhancing the image contrast of the ROI images. The image quality was expected to be better when using the image correction methods of tone curves (e.g., [13]). We hoped that the improvement in the image quality would lead to a decrease in the average error rate in liver cirrhosis classification.

In the experiments, we used grey-level transformation functions, i.e., tone curves, for image quality improvement. In this study, in order to enhance the image contrast of ROI images, we used two line-type tone curves and one curved line-type tone curve. Furthermore, we also expected that the darker regions of the ROI images would have better features as well as lighter regions. In order to implement this, we used the inverses of the tone curves. In the experiments, we also used three types of inverses of tone curves. In this paper, we examined the effect of the changes in image quality with image correction methods classifying cirrhosis of the liver on B-mode ultrasound images. The experimental results show the effectiveness of the image correction methods in improving the image quality of ROI images. By enhancing the image contrast of the ROI images, the image quality improved, and thus the generalization ability of the CNN also improved.

In the Discussion section, classical classifiers, *k*-NN (k=1,3,5), SVM, LDA, and RF, as well as the transfer learning method, VGG16, are compared to investigate the effects of the proposed method. The *k*-NN (nearest neighbour) classifier [14,15] classifies a test sample based on *k* nearest neighbour samples. The decision is basically made by *k* majority votes. In particular, the nearest neighbour classifier (k=1) is very well known and used in the pattern recognition field and in ultrasound liver classification. The SVM (support vector machine) [16] is known as an effective classifier and it is frequently used for medical imaging. The basic idea of SVM is to find a hyperplane with a margin that maximizes the distance to each sample. The LDA (linear discriminant analysis) classifier [14,15] classifies a test sample based on the statistics, mean vectors, and the same covariance matrix for each class. The RF (random forest) [17] is one of the ensemble learning approaches which are based on decision trees. The RF classifies a test sample based on the multiple outputs performed by each decision tree. The RF with multiple decision trees was expected avoid over-training. The VGG16 [18] is one of the transfer learning approaches. A VGG16 such as CNN (convolution neural network) only requires combinations of inputs or the image itself and its class label. Transfer learning was expected for a small training sample size problem such as one with the current dataset.

## 2. Materials and Methods

**Figure 3:**
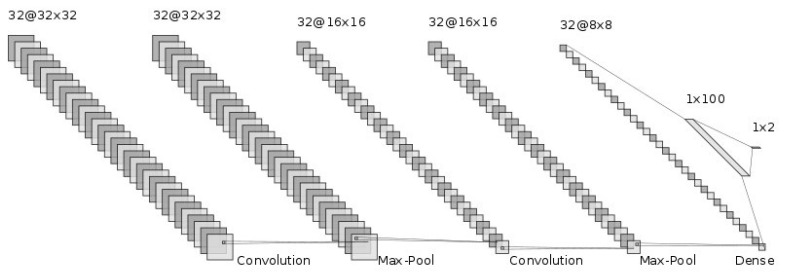
CNN structure.

The generalization ability of CNNs has been improved by adding more layers to deeply train the networks [19,20]. We hoped that the deep nets would improve the generalization ability for this difficult cirrhosis pattern recognition problem. From our preliminary experimental results, the deeper nets could memorize all the training samples. However, they seemed not to address the unknown test samples. This is known as over-training. In particular, the number of available samples, such as in this set of medical data, is limited. This is known as the small sample size problem in the pattern recognition field [21,22]. With small training sample sizes, deeper nets do not work well. Therefore, we conducted our investigation using shallow nets. The generalization ability of CNNs depends on their network structure and the parameters to be determined. First, we showed the CNN architecture, which is the same as in [6]. Figure 3 shows the structure of the CNN. The input of the CNN is the ROI image of a 32 by 32 size. Firstly, we convolved the ROI image using 32 filters with a 3 by 3 filter size. Furthermore, through 2 by 2 max-pooling, we reduced the ROI image size to a half-sized image, 16 by 16 in size. Secondly, we repeatedly convolved and performed max-pooling in the same manner. Then, we obtained 32 images 8 by 8 in size. Thirdly, we flattened this image into a 2048 (=32 by 8 by 8) dimensional vector. To implement the classification stage, we used a fully connected artificial neural network, with one hidden layer and 2 output neurons for {cirrhosis, healthy}. We explored how performance varied with the number of neurons, with 100 neurons in the final configuration, which was 2048-100-2. The intermediate activation functions were ReLU, and the output layer used softmax. The dropout rate was 0.5, with a batch size of 400. The network was optimized using ADAM over 100 time periods.

**Figure 4:**
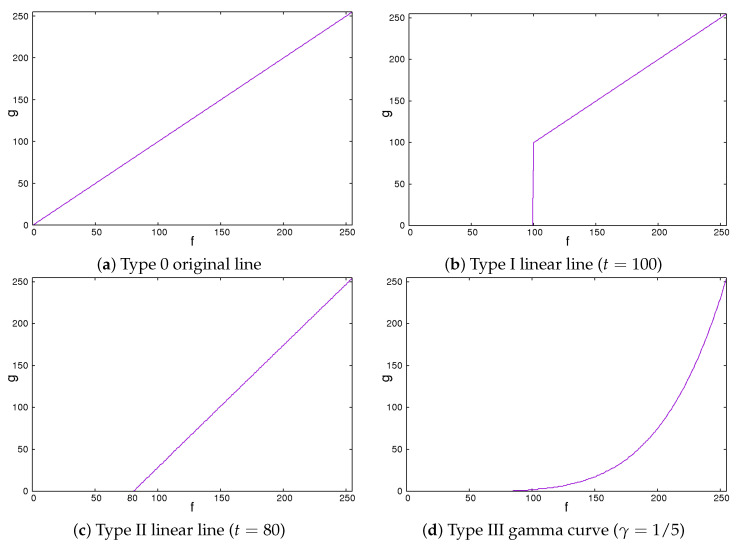
Tone curves.

**Figure 5:**
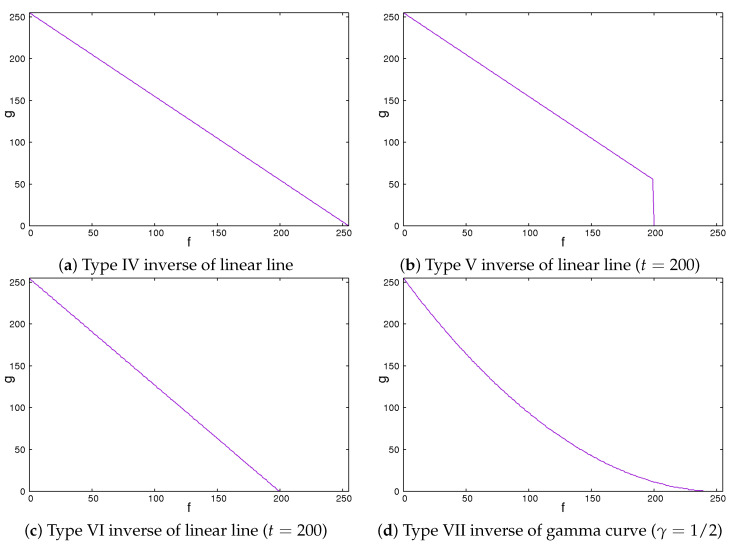
Inverse of tone curves.

Secondly, we showed the image correction methods used. We expected that the image contrast of the ROI images would be important in classifying liver cirrhosis. Thus, we adopted the approach of emphasizing the image contrast of the ROI images. In this study, we used 2 line-type tone curves and 1 curved line-type tone curve as image correction methods [13]. We also used their inverted types. Figure 4 and Figure 5 show the tone curves and the inverse tone curves. In the figures, each of the image correction processing techniques from type 0 to III corresponds to that from type IV to VII. We can see the relationships among the tone curves and the inverse tone curves. The aim of type I to III and type V to VII is to obtain enhanced-contrast images using the parameter values *t* and γ. By controlling the parameter values of *t* and γ, we expected to obtain enhanced-contrast images. The image correction techniques we used are as follows. The notation $f_{max}$ means the maximum value of the grey level. The grey level was 8. The values ranged from 0 to 255. Then, we could read $f_{max}$ as 255. In each of Figure 4 and Figure 5, the figure is drawn according to $f_{max}=255$. For 12 grey levels, the values ranged from 0 to 4095. Then, we could read $f_{max}=4095$. The notations *f* and *g* are the intensities of input and output images, respectively.

### 2.1. Type 0 Original Line

The type 0 original line is the same for both input and output values. The value of output *g* flows is the same as the input value *f*.

(1) g=f

This means that the intensities of the input and the output images are the same.

### 2.2. Type I Linear Line

The type I linear line behaves in the same manner as a linear line except for having a lower threshold point. When pixel values are less than threshold *t*, the intensity values are zero. This means they turn black and will be ignored.

(2) $$g=\left\{\begin{array}{cc} 0, & f<t\\ f, & otherwise \end{array}\right.$$

We could vary the threshold value *t* from 0 to $f_{max}$. If the value of *t* is 0, this means that the intensities of the input and the output images are the same. The higher the value of *t* is, the larger the dark regions are.

### 2.3. Type II Linear Line

The type II line is similar to the type I line. When it becomes equal to or greater than a threshold, the values of the intensity linearly increase. Otherwise, the intensities are zero.

(3) $$g=\left\{\begin{array}{cc} 0, & f<t\\ f_{max}\left(f-t\right)/\left(f_{max}-t\right), & otherwise \end{array}\right.$$

We could vary the threshold value *t* from 0 to $f_{max}$. If the value of *t* is 0, the intensities of the input and the output images are the same. These are the same as those for type I. The higher the value of *t* is, the larger the dark regions are. At the same time, the contrast of the image is enhanced.

### 2.4. Type III Gamma Curve

The gamma curve is one of the curved line-type tone curves. It is not a linear line but a non-linear curve.

(4) $$g=f_{max}{\left(\frac{f}{f_{max}}\right)}^{\frac{1}{\gamma }}$$

We could control the degree of non-linearity with the value of γ. When the value of γ is equal to 1, the intensities of the input and the output images are the same. If the value of γ is less than 1, the image is darker. Otherwise, the image is brighter.

### 2.5. Type IV Inverse of Linear Line

The type IV inverse of linear line behaves in the same manner as a linear type 0 line. The value of output *g* flows is the same as the inverse of the input value *f*.

(5) $$g=f_{max}-f$$

This means that the intensities of the input and the output images are inverse. The darker regions become lighter. By contrast, the lighter regions become darker.

### 2.6. Type V Inverse of Linear Line

The type V inverse of linear line behaves in the same manner as the inverse of a linear line except for values larger than a threshold, where the values are set to zero. That means that they turn black and will be ignored.

(6) $$g=\left\{\begin{array}{cc} f_{max}-f, & f<t\\ 0, & otherwise \end{array}\right.$$

We could vary the threshold *t* from 0 to $f_{max}$. If the value of *t* is $f_{max}$, this means that the intensities of the input and the output images are inverse. The lower the value of *t* is, the larger the dark regions are.

### 2.7. Type VI Inverse of Linear Line

The type VI linear line behaves in the same manner as the inverse of a linear line except for a greater threshold. When the pixel values are greater than the threshold, the intensity values are zero. This means that they turn black and will be ignored.

(7) $$g=\left\{\begin{array}{cc} f_{max}\left(1-f/t\right), & f<t\\ 0, & otherwise \end{array}\right.$$

We could vary the threshold *t* from 0 to $f_{max}$. If the value of *t* is $f_{max}$, this means that the intensities of the input and the output images are inverse. The lower the value of *t* is, the larger the dark regions are. Simultaneously, the contrast of the image is emphasized.

### 2.8. Type VII Inverse of Gamma Curve

The type VII inverse of gamma curve behaves as a type III gamma curve.

(8) $$g=f_{max}{\left(\frac{f_{max}-f}{f_{max}}\right)}^{\frac{1}{\gamma }}$$

We could control the degree of non-linearity with the value of γ. When the value of γ is equal to 1, the intensities of the input and the output images are inverse. If the value of γ is less than 1, the image is darker. Otherwise, the image is lighter.

In the experiments, we used ROI images modified by these image correction methods, from type I to VII and including type 0. We tried to investigate the effectiveness with and without the image correction methods on CNNs in terms of the average error rate.

## 3. Results

**Figure 6:**
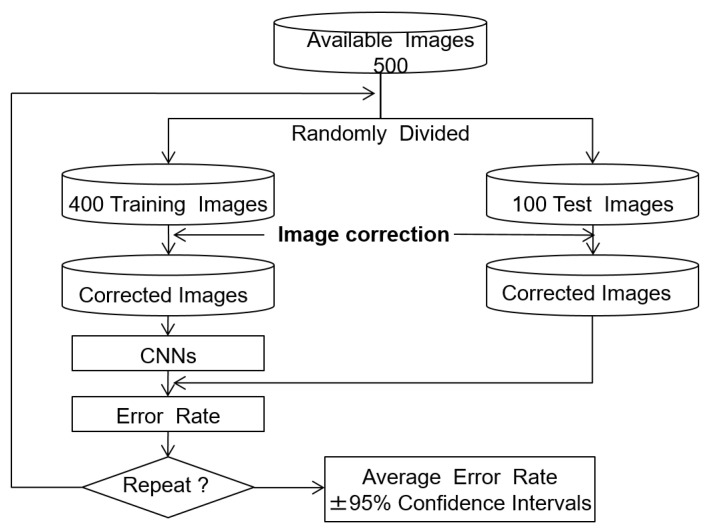
Error rate estimation with the holdout method.

The training used 500 ROI images, with 200 healthy and 300 cirrhosis examples. The effectiveness of the image correction methods was evaluated by comparing the classification error rates, which are the defined as the ratio of misclassified test images to the total number of test images. The holdout method was used to estimate the error rate, as the evaluation images were independent of the training and test images [14,15]. We report the average error rate, which gives an indication of the generalization of the classifier. The algorithm for error rate estimation is shown in Figure 6. In each trial, the 500 ROI images were split into 400 training (160 normal and 240 cirrhosis) and 100 test (40 normal and 60 cirrhosis) images. The training and test images were then modified using the tone curves as described above. The CNN was trained using the training images and the error rate was computed using the test images. This process was repeated 10 times with different random splits of the training and test images to estimate the average error rate and 95% confidence interval.

We conducted the liver cirrhosis classification experiments with tone curves and inverse tone curves. The following experiments were carried out as Experiment 1 and Experiment 2. Experiment 1 was conducted for the use of the tone curves. On the other hand, Experiment 2 was conducted for the inverse tone curves.

### 3.1. Experiment 1

The purpose of the experiment was to investigate the generalization ability of the CNN without and with tone-curved image correction methods of ROI images in terms of the average error rate. For the type I and II linear lines, the values of *t* varied from 20 to 160 every 20. For the type III gamma curve, the values of gamma ranged among 1/10, 1/5, 1/3, 1/2, 1, 2, 3, and 5. Table 1 shows the average error rates of the CNN without and with image correction methods of type I, II, and III. In the table, the upper and the lower values show the average error rate and the width of the 95% confidence interval, respectively. From the results, we chose values which gave the minimum average error rates. Then, the optimal values of type I, II, and III were t=20, t=20, and γ=1/2, respectively. Table 2 shows the details of Table 1. It provides the experimental results for values of *t* and γ. When it comes to the use of the image correction methods, type I, II, and III, we see that these methods were slightly superior to the method without image correction, type 0. In particular, the type II method gave the minimum error rate of 31.60%. It was more effective for classifying liver cirrhosis.

### 3.2. Experiment 2

The purpose of the experiment was to investigate the generalization ability of the CNN without and with inverse tone-curved image correction methods of ROI images in terms of the average error rate. For the type V and VI inverse of linear lines, the values of *t* varied from 80 to 220 every 20. For the type VII inverse of gamma curve, the values of γ ranged among 1/10, 1/5, 1/3, 1/2, 1, 2, 3, and 5. Table 3 shows the average error rates of the CNN without and with image correction methods of type IV, V, VI, and VII. From our experimental results, we selected values which gave the minimum average error rates. Then, the optimal values of type V, VI, and VII were t=160, t=200, and γ=1/2, respectively. Table 4 shows details of Table 3. It gives the experimental results for values of *t* and γ. Our experimental results showed that the average error rates of type IV, V, VI, and VII were slightly superior to those without (type 0) image correction.

From all the experimental results shown in Table 1 and Table 3, the average error rate of the type VI image correction method was the best. Its minimum average error rate was 30.60%. The *p*-value was 0.057681, compared to type 0. This means that type VI is superior to type 0 in terms of p=0.10. However, type VI is not as good in terms of p=0.01 and p=0.05.

## 4. Discussion

**Figure 7:**
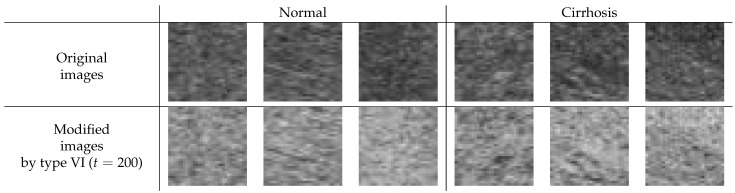
Difference between the original and the modified ROI image for normal and cirrhosis livers.

Enhancing the image contrast of ROI images is considered to lead to improved image quality, at least when considering human viewing. Here, we investigated whether the contrast enhancement would also help reduce classification error rates. Type VI (t=200) showed the best average error rate in our limited experiments. Thus, we tried to observe the modified ROI image with the image correction method of type VI. Figure 7 shows the difference between the original and the modified ROI images for normal and cirrhosis livers. From the appearance of the modified images and the experimental results, we draw the following conclusions:

1. The modified ROI images may be lighter than the original ones.
2. There seems to be a slightly enhanced image contrast in the modified ROI images compared to the original ones.

For the type VI method (t=200), the modified ROI images were much lighter because the modified ROI images were inverted and their intensities of greater than 200 were ignored. The image contrast was slightly enhanced because the gradient of the line was steeper. Through this method of image processing, we could obtain richer features for classifying liver cirrhosis. As a result, the features may be richer and the generalization ability of the CNN may improve.

On the other hand, the generalization ability of the images corrected through the type II (t=20) method yielded the second best average error rate in our limited experiments. For the type II method (t=20), the modified ROI images were much darker because the intensities of less than 100 were ignored. By cutting the darker regions and inclining the gradient of the linear line, we could obtain richer features for classifying liver cirrhosis. Thus, the features may richer and the generalization ability of the CNN may improve.

Furthermore, the effect of the classifiers which are well known and widely used as machine learning was also investigated on original images and modified images (type VI (t=200)). The classifiers were *k*-nearest neighbour, support vector machine (SVM), linear discriminant analysis (LDA), random forest (RF), and transfer learning [23,24]. The same experiments as shown in the Results section were conducted. The feature we used was the image itself. In the experiments, *k*-NN (k=1,3,5) [14,15], the linear-type SVM [16], the RF [17] with 100 decision trees, and VGG16 [18] as the transfer learning approach were used. In the VGG16, a fully artificially connected network of the same kind as previously mentioned in the CNN was added. The VGG16 was retrained by 5 out of 20 layers by fine-tuning. That is, the 15 remaining layers were frozen. Table 5 shows the average error rates of the classifiers on original images and modified images. The average error rates of the classifiers except for VGG16 were very poor. They were over 40%. There was almost no difference between the original images and the modified images. These results are in line with our previous findings [5]. In principle, these classifiers cannot avoid using one-dimensional data flattening from two-dimensional image data. They could not use the data as a 2D image. On the other hand, the VGG16 was better. A VGG16 such as the CNN could use the data as a 2D image for image pattern recognition. Slight improvement when using modified images also seems to have occurred with VGG16.

Finally, the limitations of the experiments are discussed. From the experimental results, the image correction method with type VI (t=200) for ROIs of liver cirrhosis classification on the CNN was found to work better on our dataset. When it comes to using the transfer learning VGG16, the effectiveness also seemed clear. There is a possibility for attaining an appropriate image quality through a modified method for each classifier and for each dataset. The actual selection of parameters *t* and γ for each image quality correction method should be conducted as follows, for example:

**Step 1**  Prepare several candidates for *t* or γ.
**Step 2**  Using these candidates, calculate the average error rate with available data, e.g., through the three- or five-fold cross-validation method.
**Step 3**  Select the value of *t* or γ that gives the smallest value among these average error rates.

## 5. Conclusions

In this paper, we examined the effect on the image quality of the ROI image-by-image enhancement methods on the CNN in classifying a cirrhosis of the liver on B-mode ultrasound images. The experimental results showed the effectiveness of the image enhancement methods in improving the classification of ROI images. By modifying the image contrast of ROI images, the image quality was improved and the generalization ability of the CNN improved. Thus, as the proposed method improves image quality for the pre-processing of ROI images, it is expected that the methods proposed by researchers such as [9,10,11] will also be improved. Furthermore, considering the computational cost of tone curves, we should adopt a look-up table instead of a calculation of the tone curve function. In particular, the computational cost of a non-linear function, such as a gamma correction, could be cheap. As we mentioned above, emphasizing the image contrast of ROI images is effective for improving the image quality. This can be carried out through image correction methods using tone curves. We plan to explore other types of image modification methods. One possibility is to highlight both darker and lighter regions of ROI images to improve the image quality. There are many common image correction methods. Therefore, trying these image correction methods should be considered for cirrhosis classification in future work. The validation of the proposed method for another dataset should also be addressed in future work. A CNN different from the network architecture in Figure 3 could further improve the classification performance. Furthermore, considering the network architecture of CNNs is also needed. The transfer learning approach [24] should also be considered for further improvement in liver cirrhosis classification.

## Figures and Tables

**Table 1:** Average error rates of the CNN without (type 0) and with image correction methods of type I, II, and III.

CNN (Type 0)	Type I	Type II	Type III
	(*t* = 20)	(*t* = 20)	(γ=1/2)
33.70	33.39	31.60	32.80
±4.95	±3.77	±3.78	±3.78

**Table 2:** Detail of the average error rates of the CNN with image correction methods of type I, II, and III.

Values of *t*	Type I	Type II	Values of γ	Type III
20	33.39	31.60	1/10	39.60
	±3.77	±3.78		±3.99
40	33.99	32.49	1/5	34.19
	±4.07	±4.30		±3.17
60	36.60	35.20	1/3	32.90
	±3.83	±3.82		±3.25
80	40.80	32.99	1/2	32.80
	±5.15	±3.75		±3.12
100	39.40	34.40	1	33.70
	±4.78	±2.26		±4.95
120	35.60	34.70	2	37.90
	±4.22	±2.21		±3.25
140	37.10	40.50	3	37.90
	±2.62	±3.32		±3.25
160	42.59	43.09	5	37.90
	±2.80	±3.62		±3.25

**Table 3:** Average error rates of the CNN without (type 0) and with image correction methods of type IV, V, VI, and VII.

CNN (Type 0)	Type IV	Type V	Type VI	Type VII
		(*t* = 160)	(*t* = 200)	(γ=1/2)
33.70	33.69	32.79	30.60	33.39
±4.95	±4.46	±3.26	±3.18	±3.84

**Table 4:** Detail of the average error rates of the CNN with image correction methods of type V, VI, and VII.

Values of *t*	Type V	Type VI	Values of γ	Type VII
80	42.69	42.59	1/10	38.99
	±4.35	±5.68		±4.39
100	38.79	40.40	1/5	36.10
	±4.39	±4.34		±4.13
120	37.40	37.20	1/3	35.79
	±3.58	±3.06		±4.15
140	34.50	36.20	1/2	33.39
	±4.69	±3.26		±3.84
160	32.79	34.50	1	33.70
	±3.26	±3.45		±4.95
180	33.39	33.59	2	37.90
	±4.73	±3.47		±3.25
200	32.89	30.60	3	37.90
	±4.99	±3.18		±3.25
220	33.69	31.99	5	37.90
	±4.46	±4.59		±3.25

**Table 5:** Average error rates of classifiers on original images and modified images.

	1-NN	3-NN	5-NN	SVM	LDA	RF	VGG16
	[15]	[15]	[15]	[16]	[15]	[17]	[18]
Original	45.59	44.50	45.60	44.90	43.90	45.30	36.50
images	±3.14	±3.93	±2.70	±4.20	±2.68	±4.50	±2.63
Modified images	45.89	43.89	44.90	45.59	43.39	45.20	33.00
type VI (t=200)	±5.00	±4.35	±2.60	±4.50	±3.16	±4.20	±4.03

## Data Availability

Not applicable.
